# Age Differences in the Relationship Between Daily Social Media Usage and Affect

**DOI:** 10.1093/geroni/igaa057.1320

**Published:** 2020-12-16

**Authors:** Xin Yao Lin, Margie Lachman

**Affiliations:** Brandeis University, Waltham, Massachusetts, United States

## Abstract

Social media platforms allow people to connect and share content online (e.g., Facebook, Twitter). Although older adults are becoming more frequent users of social media, there continue to be mixed views on whether social media positively or negatively impacts well-being. Past studies have mainly focused on cross-sectional analyses for individual differences. However, both the time spent on social media and one’s affect can fluctuate on a daily basis. Thus, it is important to understand how the relationship between daily social media usage and affect varies within individuals from day to day. The current study adds to the literature by examining whether daily variations in time spent with social media are related to daily positive and negative affect and whether there are age differences in these relationships. The current study used an eight-day daily diary from the Midlife in the United States (MIDUS) Refresher dataset for 782 participants (ages 25-75). Multilevel modeling results revealed that age moderated the relationship between daily time spent on social media and negative affect: for younger adults, on days when they spent more time on social media, they had more negative affect. For older adults, on days when they spent more time on social media, they had less negative affect. Surprisingly, daily time spent on social media was not related to daily positive affect, nor did this relationship differ by age. Implications for future research are discussed with a focus on how social media usage can contribute to daily well-being for adults of different ages.

